# Availability and quality of emergency obstetric care in Gambia's main referral hospital: women-users' testimonies

**DOI:** 10.1186/1742-4755-6-5

**Published:** 2009-04-14

**Authors:** Mamady Cham, Johanne Sundby, Siri Vangen

**Affiliations:** 1 Section for International Health, Institute of General Practice and Community Medicine, University of Oslo, Norway; 2 Department of State for Health, Banjul, Gambia; 3 National Resource Centre for Women's Health, Rikshospitalet Medical Centre, Oslo, Norway

## Abstract

**Background:**

Reduction of maternal mortality ratio by two-thirds by 2015 is an international development goal with unrestricted access to high quality emergency obstetric care services promoted towards the attainment of that goal. The objective of this qualitative study was to assess the availability and quality of emergency obstetric care services in Gambia's main referral hospital.

**Methods:**

From weekend admissions a group of 30 women treated for different acute obstetric conditions including five main diagnostic groups: hemorrhage, hypertensive disorders, dystocia, sepsis and anemia were purposively selected. In-depth interviews with the women were carried out at their homes within two weeks of discharge.

**Results:**

Substantial difficulties in obtaining emergency obstetric care were uncovered. Health system inadequacies including lack of blood for transfusion, shortage of essential medicines especially antihypertensive drugs considerably hindered timely and adequate treatment for obstetric emergencies. Such inadequacies also inflated the treatment costs to between 5 and 18 times more than standard fees. Blood transfusion and hypertensive treatment were associated with the largest costs.

**Conclusion:**

The deficiencies in the availability of life-saving interventions identified are manifestations of inadequate funding for maternal health services. Substantial increase in funding for maternal health services is therefore warranted towards effective implementation of emergency obstetric care package in The Gambia.

## Introduction

An overwhelming majority (99%) of the estimated 536,000 annual maternal deaths occur in developing countries making maternal mortality ratio (MMR) the indicator with the widest disparity between developed and developing countries [1]. To improve this situation, Millennium Development Goal 5 targets a three-quarter maternal mortality reduction by 2015 [2]. Unrestricted access to high quality emergency obstetric care (EOC) is promoted to the attainment of that goal [3]. EOC and skilled attendance at delivery are two complimentary strategies closely correlated with MMR [4-6]. Countries with low MMR, such as those in Europe and North America, have both a high proportion of births attended by skilled provider and universal access to high quality EOC [4-6]. By contrast, in many developing countries both the proportion of births attended by skilled personnel and met need for EOC are disproportionately low [5,7,8]. The latter is indeed the situation of Gambia where the present study was performed [9].

Gambia's maternal health policy puts emphasis on referral to tertiary hospitals for high-risk pregnancies with the goal to reduce maternal and perinatal morbidity and mortality. Geographical accessibility to health care facilities in the country is good with over 85% and 97% of the population living within 3 km and 5 km of a primary health care or outreach health post respectively [10]. Cost of maternity care services in public health facilities is relatively low with a one-time standard fee of five Dalasis (abbreviated D, US$ 1 equivalent to D26 as at September 2006) payable on antenatal registration. Institutional normal vaginal delivery and cesarean section attracts an additional official fee of D50 and D100 respectively [11]. Payment of these fees should cover drugs, medical supplies, overnight admission and other services including blood transfusion during labor, delivery and immediate postpartum period. There is no required payment of fees in advance of admission or care. However, it is not uncommon for patients be handed prescriptions only to buy items when not available in the hospital.

With all these efforts, Gambia's maternal health indicators are not favorable. Maternal mortality ratio, for example, is up to 1500 per 100000 live births and lifetime risk of maternal death is over 200 times higher than in developed countries [1]. Most deliveries (70%) occur at home supervised by a traditional birth attendant or a relative and only one in five women with obstetric emergencies report to a medical facility for assistance [9]. Thus, a great proportion of women requiring life-saving obstetric services do not get to such services.

Understanding the factors that hinder optimal utilization of available maternity care services particularly when an emergency complication arises is essential in addressing the barriers and to substantially reduce maternal mortality. Exploring women-user's experiences with the health system is thought to be most appropriate. This approach has been reported to have increased both acceptability and utilization of obstetric services elsewhere [12,13].

Investigations into maternal health care have often used maternal deaths as a starting point. Review of severe acute maternal morbidity (SAMM) cases has now been proposed as an entry point. Besides being far more common, SAMM cases, unlike maternal deaths, are women who have survived and they, rather than their family members, can be interviewed about care seeking efforts and detailed aspects of the care received [14-16].

This paper reports on findings from a qualitative study of women survivors of SAMM treated at Gambia's main referral hospital. The study explored the process of seeking and obtaining obstetric care services with the aim to assessing round the clock availability and quality of EOC services. Special attention was given to possible barriers to accessing the required care.

## Methods

### Study country

Located in West Africa, Gambia has a population of 1.4 million inhabitants, mainly subsistent farmers. Of 177 countries on the Human Development Index for 2006, The Gambia was classified as a low-income country and ranked 155^th ^[17]. The gross national product per capita is $340. Though resource-constraint, public spending on the health sector continuously increased over the years, currently accounting for 13.9% of government spending, being ranked the second highest in the African Region [18]. However, the proportion of government expenditure specific to maternal health remains unknown. Health has been identified as a priority by the Gambian government and there is great enthusiasm to attain Millennium Development Goals on child and maternal health which has culminated in 2005 the development of a country road map to reduce maternal and neonatal morbidity and mortality [19]. Sadly, for lack of funding this road map is yet to be implemented.

### Study hospital

Royal Victoria Teaching Hospital (RVTH), the site for the current study located in the capital city Banjul, was selected purposively for being the main obstetric referral hospital in the country and with an overwhelming majority of the country's health resources. For example, almost all doctors and 45% of midwives in the public sector work at RVTH [20]. It has a separate operating theatre exclusively for maternity cases with up to three teams of four doctors (a consultant obstetrician and three residents) supposedly to provide round the clock obstetric services cover. However, only few junior doctors are available after normal working hours (8:00 – 14:00 hours) and on weekends. EOC service in the hospital is supported by the hosting of the National Reference Laboratory which includes the National Blood Transfusion Services. Unlike other public hospitals around the country, electricity and water supply at RVTH is available round the clock. With these and other facilities, it is widely believed that EOC services at RVTH are more readily available and of superior quality than in other public hospitals. Thus it is not surprising that 35% of births in medical facilities and 79% of cesarean sections performed in the country occur in RVTH [9]. Besides its primary function being an obstetric referral center, RVTH also provides general pregnancy care services to women living within close surroundings. The MMR at this hospital is very high, exceeding 1100 per 100,000 live births [21].

### Subjects and data collection

In-depth interviews with women survivors of severe acute obstetric complications or "Near Misses" were held. SAMM case was defined as "any woman who suffered acute obstetric conditions, at any period in pregnancy to six weeks postpartum, severe enough to end in a maternal death. The woman survived due to the care received or good luck"[15]. We included five categories of obstetric emergencies defined according to disease-specific criteria based on management and/or clinical signs and symptoms: hemorrhage at any pregnancy state (leading to transfusion, cesarean section or hysterectomy); hypertensive pregnancy disorders including eclampsia or severe pre-eclampsia with a minimum diastolic pressure of 110 mmHg; puerperal sepsis (peritonitis, septicemia, offensive vaginal discharge); dystocia resulting from prolonged, obstructed labor or mal-presentation (leading to ruptured or pending uterine rupture, cesarean section, instrumental delivery or perinatal laceration) and severe anemia (hemoglobin < 6 g/dl). The lower limit of diastolic pressure and hemoglobin level applied were according to national guidelines [22].

To appreciate round the clock EOC availability, we purposively selected 30 women from weekend admissions between January and June 2006. We ensured inclusion of all the above obstetric conditions. For budgetary reasons and feasibility, only women residing within 30 km of the hospital were recruited which translates to residents of three urban municipalities: Banjul, Kanifing and Western region. Individual consent and women's telephone contacts and traceable addresses were obtained before discharge from the hospital. Interviews were conducted at the women's homes and convenience within two weeks of discharge in the presence of relative(s) who were with her in hospital. The primary author (MC) with local experience performed all the interviews in the local languages which focused on health care seeking process, woman's experience at the hospital from arrival to discharge, estimated time lapse between reception and obtaining definitive treatment. The woman's perceived quality of care received was also explored. Interview guides were semi-structured, open-ended and probing that permitted women to respond freely using their own language. All interviews were transcribed verbatim, translated into English, categorized and analyzed using a Grounded Theory [23]. The frequently emerging themes and concepts were organized accordingly with the aim of identifying pertinent issues of relevance during care seeking and obtaining process. Typical statements were used for citation. Interview reports were supplemented by quantitative data on the number and types of obstetric condition or event each woman had, management and treatment received with their timing abstracted from multiple maternity data sources including case files, theatre and blood transfusion registers and ward daily report books. Ethical approval for this study was obtained from the ethics committees in both Gambia and Norway.

## Results

### Characteristics of women

Mean age of the women was 24 years with a wide range (17–38 years) and average previous number of deliveries was two. All except four women were married, 11 attended formal schooling with six completed 11 years of secondary school. Antenatal care visit was noted for all except two women with a reported average number of three visits (range 1–7). Direct obstetric complications were the primary diagnosis for 25 women: hemorrhage (n = 6); hypertensive pregnancy disorder (n = 12 of which eclamptic seizures noted in five); dystocia and sepsis accounted for five and two cases respectively. Severe anemia was noted in five women. Multiple conditions were noted in four of the 30 women. All except two women sought medical help initially from a nearby health center before further referral to the study hospital. Of the 22 women that gave birth two had twins. Twenty of the women delivered at the study hospital with ten of them delivered by cesarean section. Six women experienced a stillbirth.

Estimated time interval between diagnosis and initiation of definitive treatment varied by condition or management. Women who received blood transfusions, Magnesium Sulphate (MgSo4) or had cesarean section, were associated with considerable longer delays with a reported average time of 48 hours (ranged 5 – 72 hrs), 12 hrs (ranged 4 – 48 hrs) and 24 hrs (ranged 2 – 72 hrs) respectively.

We noted wide variation in reported treatment cost and scheduled fees even when final cost calculation was limited to financial costs on admission, drugs, medical supplies and transfusion blood. The average total expenditure for these women was between 5 and 18 times higher than the standard fees (table 1). Substantial variation in treatment costs by condition or management was also noted adding to the unpredictability of the final costs. The average total expenditure for women receiving transfusion or treated for hypertensive pregnancy disorder compared to women who did not receive such treatment was D881, D586 and D234 respectively, indicating a sharp difference.

**Table 1:** Expenditures by treatment of obstetric condition

**Condition**	**Expenditures in Gambian Dalasis – mean costs (range)**		
	
	**Admissions fee**	**Drugs and/Blood**	**Total**
Standard charges	50 (50 – 100)	-	50 (50 – 100)
Transfused cases only	346 (50 – 1500)	512 (70 – 1500)	881 (180 – 2200)
HPD cases only	245 (50 – 1500)	425 (0 – 800)	586 (100 – 1950)
Transfused/HPD cases	316 (50 – 1500)	390 (0 – 1500)	765 (100 – 2200)
Not Transfused or Treated for HPD	156 (100 – 225)	61 (0 – 200)	234 (100 – 400)
All cases	291 (50 – 1500)	341 (0 – 1500)	687 (100 – 2200)

### Reception at the referral hospital

For some women even with a referral letter obtained from a peripheral health unit, reaching the hospital in an emergency state had not resulted in receiving prompt care. Women waited for hours before being formally received or attended to at the hospital. Narratives of an escort to a 17-year-old eclamptic woman provide a poignant case:

*"She fell down unconscious at home.......we (me and her boyfriend) took her to the nearest health center.........there she was examined and put in the ambulance to Banjul (RVTH). On our arrival at the hospital the security man told me to first go to the delivery room to inform the nurses..... I went and told them (nurses and midwives) but I was told to "go and wait". They never came to see her (patient). An hour later, when she again started fitting I went back to tell them. That was the moment one of the nurses came to see her. Thereafter she was wheeled to the ward and put on a bed"*.

### Obtaining blood and blood transfusion

Availability of safe blood for transfusion is essential for effective implementation of EOC package. Thus, in situations were blood is not readily available prompt delivery of life saving interventions such as transfusion or cesarean section is hindered. Receiving definitive obstetric care was for some women, constrained by many impediments surrounding blood transfusion. Lack of transfusion blood in the hospital emerged as an important factor. The husband of a severely anemic multi-parous woman needing transfusion narrated how he acquired blood:

*".....At the ward I was told to find two bottles of blood for her (referring to his wife). I went to the lab to look for blood but was told (by laboratory staff) that there was none......I decided to donate but my blood (group) was different. It was already night so I went home..........In the evening of the following day a friend came to donate her one bottle"*.

Obtaining the requested quantity of blood was both taxing and time consuming. However, the process highlighted the crucial role husbands played in acquiring transfusion blood. For some, blood was acquired mostly through social networks at no financial cost after a long period of searching for a potential donor far and wide. A husband-escort narrated:

*"A whole day I cannot find a donor or get blood. The following morning I went straight to the army headquarters (next to the hospital) to seek for help. Luckily one of the soldiers followed me to the lab and volunteered to donate one bottle. The second bottle was donated by a friend of mine (living 70 km away) the next day"*.

Making blood readily available to patients needing transfusion should supposedly be the primary function of the hospital. But similar to other hospitals around the country, in RVTH this responsibility was shifted to the women and their relatives. None of the 15 women transfused in the current study obtained blood from the hospital's regular supplies. These women obtained transfusion blood from a combination of ways including from a relative (n = 6) free of cost, a remunerated donor identified by laboratory staff or by directly buying from laboratory staff (n = 13) even when buying and selling of blood in public hospitals is prohibited. The cost per unit of blood varied (range D200 – D350). The costs incurred on blood were strikingly prohibitive as a husband narrated:

*"......I was told to find seven bottles of blood for her (referring to his wife). I donated one.... (after a long paused in hesitation to explain) ..... my friend accompanying me talked to one guy working in the laboratory to seek for assistance. He demanded D250 for each bottle....... I paid him D1500 for the six bottles"*.

Even with the multiple hurdles associated with acquiring blood surmounted, other challenges remain. Inadequate storage of transfusion blood by ward staff prevented at least one woman from being transfused. A 27 year-old woman with severe obstetric hemorrhage painfully recalled how she could not be transfused.

*"My husband managed to buy two bottles of blood for me yesterday. The morning ward staff collected the blood from the lab and put them on top of the ward refrigerator for cooling. The following morning my husband was again told to replace the two bottles as the previously acquired blood was spoiled as the nurse put it"*.

### Obtaining cesarean section

Cesarean section, an essential component of the EOC package, provided in a timely fashion is critical for maternal and fetal outcomes. Health service inadequacies particularly shortage of doctors prevented timely dispensation of cesarean delivery. For some women in the current study, despite the urgency, cesarean section was delayed for four days after the recommendation was made. Testimonies of the mother-in-law of an eclamptic woman needing cesarean section provide a classic example:

*".....her legs and face were swollen and fitting throughout. On Friday she was transferred to the hospital in Banjul (RVTH). I was told she will be operated (cesarean section) when the doctor is available........She was operated on Monday (three days later) but the baby was already dead"*.

### Impact of essential drugs' shortage

Intermittent shortage of essential medicines especially MgSo4 in the hospital was an important factor for suboptimal care. Given the high costs of medicines in privately owned pharmacy stores meant that in most cases family members of the women were unable to buy all the prescribed medicines unavailable in the hospital. Furthermore, the few that managed could hardly afford the full course of treatment ordered. This was a pervasive recurrent issue raised as indicated in this testimony of a husband of a woman with eclampsia:

*".....They wrote three different types of medicines for her and none was available in the hospital. As I had no money with me I went back home to look for more money. It was not until the following day that I raised some money enough to buy only one of the medicines prescribed"*.

### Perceived quality of care received

Women's testimonies depicted mixed reactions. As some expressed satisfaction with the quality of care received others did not. Interpersonal care processes such as being greeted or talked to, bed sheet frequently changed or medications provided free of cost were commonly cited by women who perceived the quality of care received satisfactory. Survival of the illness even when the pregnancy outcome was fetal loss was associated with good quality of care. In contrast, poor reception, unpleasant provider attitude, difficulties encountered in acquiring blood and actual transfusion were omnipresent concerns in the accounts of women who perceived the quality of care below expectation. The higher than expected costs of treatment were sources of indignation, shock and disappointment to women and their families as exemplified by this quote from a husband:

*"We are told maternity fees are not more than D100. We go to the hospital with only that amount. In reality there is nothing in the hospital........ You are asked to buy blood, medicines and other things. If you don't have money your patient will die. I spent D2200. I spent all that I have and even borrowed money to meet the total cost"*.

## Discussion

This study uncovered substantial difficulties in obtaining EOC services in Gambia's main referral hospital. Health service related inadequacies resulting from a *"plethora of shortages" *including lack of transfusion blood, shortage of essential medicines especially MgSo4 and shortage of doctors underscored the obstacles. These shortages had a negative impact on timely access to the required obstetric care.

The acute shortage of blood for transfusion in this hospital is worrying given that availability of blood is essential in treating common obstetric conditions including hemorrhage, anemia and for at least 6.4% of women who needed cesarean section [24]. Anemia and hemorrhage are the leading causes of maternal mortality in this hospital accounting for over half of all deaths, with anemia related deaths alone increased by six-fold between 1991 and 2001 [21]. Given the strong association between maternal mortality and blood availability [25], ensuring local availability of blood for prompt access to transfusion when required is therefore warranted. Despite being reported previously [26-28], scarcity of transfusion blood in Gambian hospitals still persists. In our opinion this situation is self-reinforcing and driven by a combination of factors including the general fear of testing positive for HIV in a population where HIV is highly stigmatized. This could cause potential donors are reluctant to come forward to donate blood. Additionally, the high anemia prevalence (52%) among Gambian women of reproductive-age [29] increases the need for transfusion blood. Thus, the demand far exceeds the supply causing consistent shortages. Importantly, transfusion blood in public hospitals around the country including RVTH is mostly acquired from directed donors. Most disquieting situation is the reliance on commercial donors for blood availability. Besides yielding a lower donation rate, remunerated blood donation increases the risk of transfusion transmitted infections [30,31]. Therefore, to meet the country's blood transfusion needs, the national blood transfusion services must take a more proactive role in promoting voluntary donation. In addition to substantial maternal mortality reduction, local blood availability may have some ripple effects in benefiting non-obstetric patients, facilitating speedy dispensation of care and for overall utilization of services [32].

A striking finding of this current study is the huge costs involved in obtaining required obstetric treatment. Had time and other indirect costs on travel, food, living in the hospital and caretaker been included in the calculation, the final costs would have been even larger. These expenses form a substantial part of health care costs elsewhere [33-36]. Of interest is the fact that more than two-thirds (68%) of the costs were indirect costs such as for transfusion blood, MgSo4 and medical supplies that the hospital lacks. These shortages highlight the operational difficulties in this national hospital but to a broader context mirror inadequate health system funding particularly for Gambia's maternal health program. This is evidenced by the current lack of budget for maternal health services even with the reported increased spending on health. Thus, the shortages identified in this study are not unexpected.

Private funding constitutes 60% of Gambia's total expenditure on health with 67% derived from user-fees [18]. Government's spending on health per capita is currently at $8 rather than the required $12 to provide minimum level of health services [18]. In 2007 the government, however, abolished all user-fees on maternity care services. Unfortunately, this move has not yet culminated in increased funding to replenish the lost revenue from such fees creating an income gap for the health sector. Given this situation, it is reasonable to conclude that the health service deficiencies identified in this study may not be adequately addressed at least for now. Therefore, along with abolishing user-fees there should be increased investment. That would be a more sustainable and sound approach in providing financial protection to women and their families.

Though our data is limited in determining affordability of treatment cost, we noted that for almost all women their accompanying family or friends did not have the needed cash in hand to pay for the prescribed drugs or blood donor. Instead they went back to their homes to raise more money, a process that deferred access to definitive treatment. This may have serious implications for maternal and fetal health outcomes. The relatively short window period for treating hemorrhage, for example, a delay of 12 hours [37] could be catastrophic. The average delay of 48 hours before the actual initiation of blood transfusion as found in this study provides clear indication of the substandard quality of EOC in this hospital. This could possibly explain the high maternal mortality associated with hemorrhage and anemia as reported in this hospital [21]. The high and unpredictable treatment costs uncovered makes it more difficult to save money for emergency care and may potentially serve as a strong deterrent to seeking future obstetric care even in emergency situations particularly among poor women [35,36].

Although, considering users' view in determining quality of care has been criticized [12,38], however, its measurement fulfils important issues of care including: understanding users' experiences of health care, promoting cooperation, identifying problems and evaluating health care [38]. The findings of this study show that there are inadequacies that resulted in significant delays before receiving definitive treatment. Substantial fetal losses (one in three women experienced a stillbirth) were noted. Fetal outcomes and quality of obstetric care are closely correlated [39]. Additionally, the mean hospital stay of 10 days obtained compares unfavorably with the Irish study [40]. This may to some degree reflect poor quality of services. Poor quality of care, perceived or real, has been reported to result in delayed care seeking and poor outcomes [41].

Poor staff attitudes that emerged in women's accounts in this study are not surprising as it has been reported by previous studies [10,13,26,36,42-44]. We speculate that instituting a system of perinatal audit in this hospital's maternity wing would continually improve obstetric care services and maternal health outcomes.

This hospital based study on a sample of women with different conditions highlighted their varied but real individual experiences in obtaining EOC at Gambia's main obstetric referral hospital. Their accounts of events may have been less prone to recall bias given the short recall period. Conducting interviews in participants' homes may have considerably minimized possible courtesy bias, moreover, when the interviewer was neither known nor connected to the study hospital. Involvement of key people who where with the woman at the hospital in the interviews provided detailed information about events. In our opinion the above approaches applied strengthened the current study and impacted favorably on the validity of our findings.

## Conclusion

This study highlighted substantial inadequacies in the availability and quality of life-saving obstetric services in RVTH, which impacted on timely receiving of urgently needed obstetric care. These inadequacies are manifestations of a broader health system problem but most importantly mirror inadequate health care funding. In order to improve availability and quality of obstetric care services in the country and RVTH in particular, there is an urgent need for substantial increased and sustained funding for maternal health services. Along with that we suggest that instituting a system of perinatal audits in this hospital is a necessary intervention. This would continuously improve the quality of obstetric care services and may possibly reduce the high maternal mortality rate in this hospital.

## Key Points

Prompt access to high quality emergency obstetric care (EOC) is not only essential for positive maternal and fetal outcomes – it is also critical for future utilization of such services.

• Women seeking EOC services endure substantial delays before receiving definitive treatment;

• Multiple health service factors including lack of transfusion blood, shortage of essential drugs (particularly Magnesium Sulphate) and shortage of doctors contributed to these delays;

• The need for transfusion or treatment of hypertensive pregnancy disorders inflated the final treatment costs to up to 18 times greater than the standard fees;

Inadequate funding for the health system played an important role. Therefore, increased investment in the health system may substantially improve the quality of EOC services.

## Abbreviations

EOC: Emergency Obstetric Care; MMR: Maternal Mortality Ratio; Magnesium Sulphate: MgSo4; RVTH: Royal Victoria Teaching Hospital; SAMM: Severe Acute Maternal Morbidity

## Competing interests

The authors declare that they have no competing interests.

## Authors' contributions

MC conceived of the study, did the data collection and analysis, wrote the first draft and final paper. SV participated in the draft of the manuscript and final paper. JS supervised the project, reviewed the first and final paper. All authors read and approved of the final manuscript.
